# Analyzing Image Segmentation for Connectomics

**DOI:** 10.3389/fncir.2018.00102

**Published:** 2018-11-13

**Authors:** Stephen M. Plaza, Jan Funke

**Affiliations:** Howard Hughes Medical Institute, Ashburn, VA, United States

**Keywords:** image segmentation, evaluation, metrics, connectomics, electron microscopy

## Abstract

Automatic image segmentation is critical to scale up electron microscope (EM) connectome reconstruction. To this end, segmentation competitions, such as CREMI and SNEMI, exist to help researchers evaluate segmentation algorithms with the goal of improving them. Because generating ground truth is time-consuming, these competitions often fail to capture the challenges in segmenting larger datasets required in connectomics. More generally, the common metrics for EM image segmentation do not emphasize impact on downstream analysis and are often not very useful for isolating problem areas in the segmentation. For example, they do not capture connectivity information and often over-rate the quality of a segmentation as we demonstrate later. To address these issues, we introduce a novel strategy to enable evaluation of segmentation at large scales both in a supervised setting, where ground truth is available, or an unsupervised setting. To achieve this, we first introduce new metrics more closely aligned with the use of segmentation in downstream analysis and reconstruction. In particular, these include synapse connectivity and completeness metrics that provide both meaningful and intuitive interpretations of segmentation quality as it relates to the preservation of neuron connectivity. Also, we propose measures of segmentation correctness and completeness with respect to the percentage of “orphan” fragments and the concentrations of self-loops formed by segmentation failures, which are helpful in analysis and can be computed without ground truth. The introduction of new metrics intended to be used for practical applications involving large datasets necessitates a scalable software ecosystem, which is a critical contribution of this paper. To this end, we introduce a scalable, flexible software framework that enables integration of several different metrics and provides mechanisms to evaluate and debug differences between segmentations. We also introduce visualization software to help users to consume the various metrics collected. We evaluate our framework on two relatively large public groundtruth datasets providing novel insights on example segmentations.

## 1. Introduction

The emerging field of EM-level connectomics requires very large 3D datasets to even extract the smallest circuits in animal brains due to the high resolution required to resolve individual synapses. Consequently, at typical nanometer-level resolution single neurons in even a fruit-fly brain typically span over 10,000 voxels in a given orientation. An entire fly dataset which is less than 1*mm*^3^ requires over 100TB of image data (Zheng et al., 2017).

**Figure 1:**
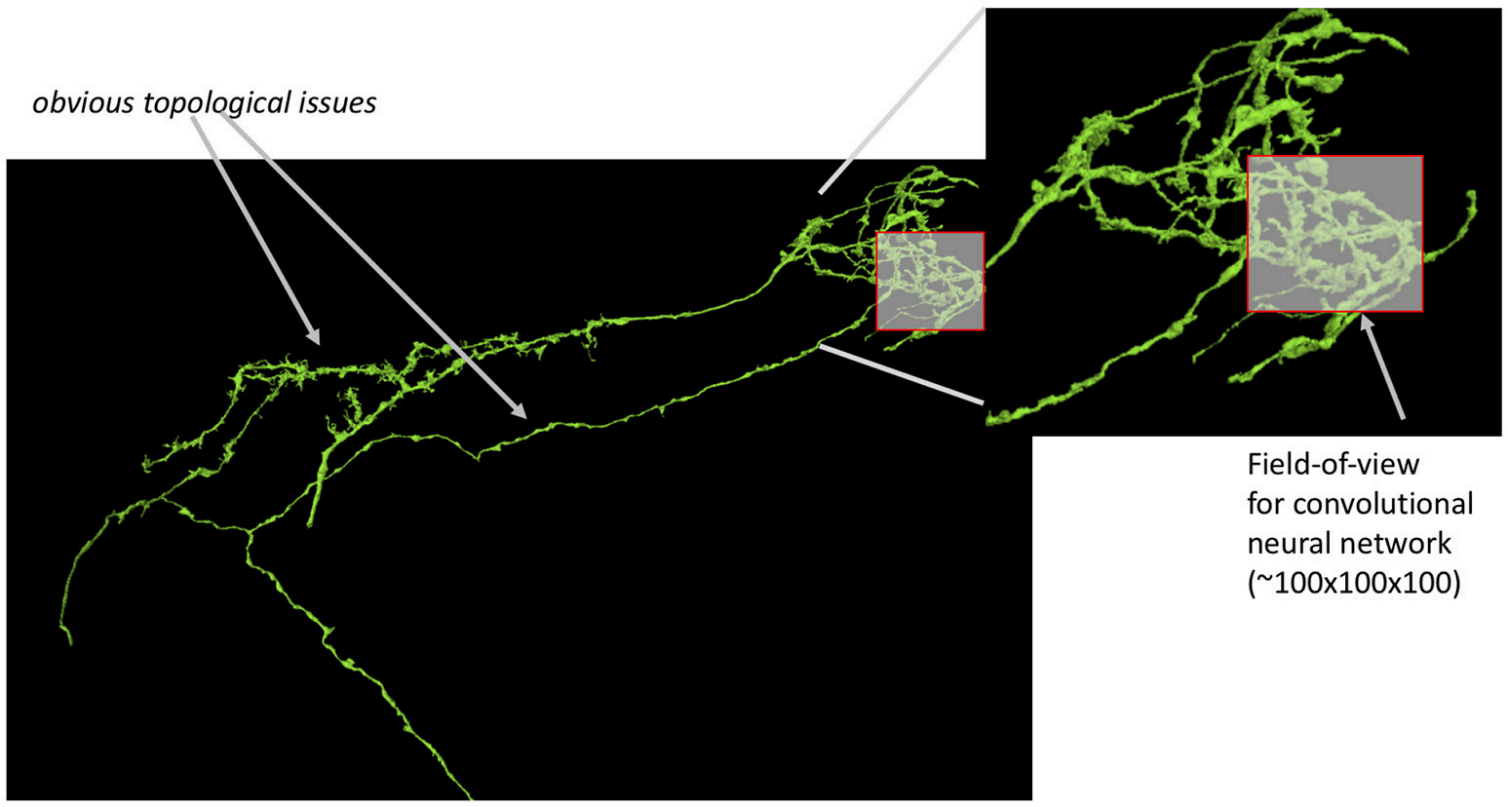
Small segmentation errors locally can lead to large topological errors. The field-of-view for modern convolutional neuronal networks is a small fraction of the size of the neuron leading to potentially bad global mistakes. Segmentation evaluation is typically done on datasets only a few times bigger (in one dimension) than this field of view.

**Figure 2:**
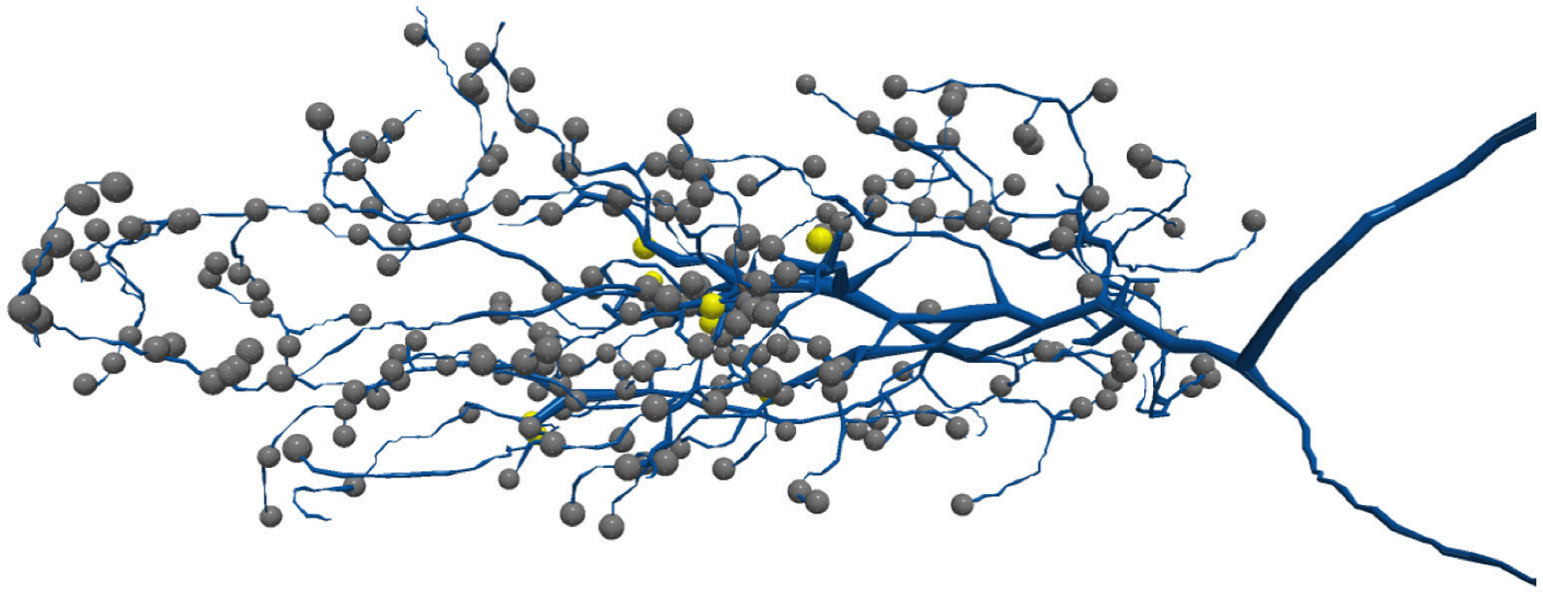
Synapses are often located on thin neurites. The above shows a T5 neuron where many synapses are on the neuron tips. Each sphere represents a different synapse site.

These dataset sizes pose several challenges for automatic image segmentation, which aims to automatically extract the neurons based on electron-dense neuron membranes. First, image segmentation algorithms struggle with classifier generalizability. For a large dataset, there are greater opportunities for anomalies that are significantly outside of the manifold of training samples examined. Even with advances in deep learning (Funke et al., 2018; Januszewski et al., 2018), the size and high-dimensional complexity of neuron shapes allow even small segmentation errors to result in catastrophically bad results as shown in Figure 1. Independent of dataset size, image segmentation struggles in regions with image contrast ambiguity, inadequate image resolution, or other image artifacts. This is particularly prominent for small neurites where synapses often reside (Schneider-Mizell et al., 2016). In Figure 2, the synapses for the neuron reside on the small tips of the neurons.

It should follow that image segmentation should be evaluated on large datasets with additional consideration for the correctness of small neurites critical for connectivity. Unfortunately, this is not the case. The authors are aware of no publications for new segmentation algorithms that emphasize this. Recent work (Maitin-Shepard et al., 2016; Januszewski et al., 2018) have evaluated segmentation on large datasets, such as Takemura et al. (2015). But these works do not consider synaptic connectivity explicitly, which is the ultimate application of the image segmentation. Neither SNEMI (Arganda-Carreras et al., 2015) nor (CREMI, 2016) segmentation challenges use datasets that span large sections of neurons. While they have been instrumental to meaningful advances to the field, they are ultimately limited by their small size and can under-represent problems as shown in Figure 1. This occurs because the actual cause of the error is in only one small region, but the impact is observed in many more regions.

There are reasons large-scale, connectivity-based evaluations are uncommon. Importantly, evaluating large datasets requires considerable ground truth that is time-consuming to produce. The groundtruth dataset in Takemura et al. (2015) is an order-of-magnitude bigger than the other public challenges but took 5 years of human proofreading and is still over three orders of magnitude smaller than the whole fly brain. We believe connectivity-based metrics have not been readily adopted because (1) it requires the annotation of synapse objects which is an independent step of the typical segmentation workflows, (2) the segmentation optimization objectives used in classifier training focus on lower-level, local topology (Rand, 1971; Meilă, 2003), whereas connectivity is more global, (3) there are no proposed connectivity metrics that are widely adopted, and (4) there are no sufficiently large challenge datasets to meaningfully capture neuron connectivity. For algorithm designers, it is probably disconcerting to achieve poor evaluation scores based on connectivity that cannot be directly optimized in segmentation objectives without clever engineering and heuristics. While the training and local validation of segmentation is both practical and leading to significant improvements to the field, ignoring the higher-level objectives could lead to an over-estimation of segmentation quality and missed opportunities for more direct improvements for the target applications. We will show later that evaluating segmentation around synapses more directly results in less optimistic scoring compared to traditional metrics like (Meilă, 2003). Recent work in Reilly et al. (2017) also introduced a metric that more appropriately weighs the impact of synapses on segmentation, though it does not explicitly consider connectivity correctness between neurons.

To address these issues, we propose a segmentation evaluation framework, which allows one to examine arbitrarily large datasets using both traditional and newly devised application-relevant metrics. Our contributions consist of (1) new evaluation metrics, (2) novel mechanisms of using metrics to debug and a localize errors, (3) software to realize these evaluations at scale, and (4) visualization to explore these metrics and compare segmentations.

We advocate an “all-of-the-above” philosophy where multiple metrics are deployed. In addition, we provide an approach to decompose some of these metrics spatially and per neuron to provide insights for isolating errors. This overcomes a limitation in previous challenge datasets that mainly produce summary metrics over the entire dataset, which provides no insight to where the errors occur. By decomposing the results, our framework is useful as a debugging tool where differences between segmentations are highlighted. While ground truth is ideal for evaluating different segmentations to know which one is better in an absolute sense, these debugging features highlight differences even if directly comparing two test segmentations without ground truth. This is critical for practically deploying segmentation on large datasets. The best segmentation can often be discerned by quickly examining the areas of greatest difference. While this provides only a qualitative assessment, this information is useful for identifying areas where new training data could be provided. Also, if one samples some of these differences, potential impact on proofreading performance can be discerned. For instance, such analysis might reveal that the most significant differences are due to one segmentation having a lot of large false mergers, which tend to be time consuming to fix.

Beyond decomposing metrics in new ways, we introduce the following evaluations:

- A novel, synapse-aware connectivity measure that better encapsulates the connectomics objective and provides intuitive insight on segmentation quality.
- New strategies to assess segmentation quality with different definitions of connectome completeness, 95 providing a potentially more lenient and realistic optimization goal. This is motivated by research that suggests a 100% accurate connectome is unnecessary to recover biologically meaningful results (Takemura et al., 2015; Schneider-Mizell et al., 2016; Gerhard et al., 2017).
- Ground-truth independent statistics to assess segmentation quality, such as counting “orphan” fragments and self-loops in the segmentation. These statistics provide additional mechanisms to compare two segmentations without ground truth.

The above is deployed within a scalable, clusterable software solution using Apache Spark that can evaluate large data on cloud-backed storage.

We evaluate this ecosystem on two large, public datasets. Our parallel implementation scales reasonably well to larger volumes, where a 20 gigavoxel dataset can be pre-processed and evaluated on our 512-core compute cluster in under 10 min with minimal memory requirements. The comparison results emphasize the importance of considering the synapse connectivity in evaluation. We also show that groundtruth is not necessary to generate interesting observations from the dataset.

The paper begins with some background on different published metrics for segmentation evaluation. We then introduce the overall evaluation framework and describe in detail several specific new metrics. Finally, we present experimental results and conclusions.

## 2. Background

Several metrics have been proposed for segmentation evaluation, where the goal is analyzing the similarity of a test segmentation *S* to a so-called ground truth *G*. We review four categories of metrics in this section: volume-filling or topological, connectivity, skeleton, and proofreading effort.

### 2.1. Volume-filling or topological

Topological metrics measure segmentation similarity at the voxel-level, so that the precision of the exact segmentation boundaries is less important than the topology of the segmentation. For instance, if the segmentation splits a neuron in half, the similarity score will be much lower than a segmentation that mostly preserves the topology but not the exact boundary. Example metrics of this class include the Rand Index (Rand, 1971; Hubert and Arabie, 1985), Warping Index (Jain et al., 2010), and Variation of Information (VI) (Meilă, 2003). Since VI will be discussed later in this work, we define it below as:

(1) $$\begin{array}{r} VI\left(S,G\right)=H\left(S|G\right)+H\left(G|S\right) \end{array}$$

where H is the entropy function. VI is decomposed into an over-segmentation component *H*(*S*|*G*) and an under-segmentation component *H*(*G*|*S*). A low score indicates high similarity.

### 2.2. Connectivity

Examining topological similarity using the above metrics can be misleading in some cases since small shifts in segment boundaries can greatly impact the scores as noted in Funke et al. (2017). Furthermore, as shown in Figure 2, the synaptic connections are often on the harder-to-segment parts of a neuron that only make a small percentage of overall neuron volume. One potential solution is to define *S* and *G* in Equation 1 over a set of exemplar points representing synapses, instead of all segmentation voxels as done in Plaza (2016) and Plaza and Berg (2016). A similar strategy of measuring groupings of synapses was introduced in Reilly et al. (2017), which additionally breaks down results per neuron making the results more interpretable. While these metrics better emphasize correctness near synapses, it is not obvious how to interpret error impact to connectivity pathways.

### 2.3. Skeleton

Similar to topological metrics, the works in Berning et al. (2015) and Januszewski et al. (2018) describe metrics based on the correct run-length of a skeleton representation of a neuron. This class of metric provides an intuitive means of interpreting data correctness, namely the distance between errors. In (Berning et al., 2015), the run length can be very sensitive to small topological errors if one tries to account for synapse connectivity since synapses can exist in small neuron tips or spine necks where segmentation errors are more prevalent due to the small size of the processes. While this can be useful to emphasize synaptic-level correctness, it can also under-value a neuron that is mostly topologically correct. Januszewski et al. (2018) proposes an expected run length metric (ERL) that proportionally weights contiguous skeleton segments. While ERL is the most topologically intuitive metric, it conversely suffers from under-weighting correctness for small process such as at dendritic neuron tips in *Drosophila* or spine necks seen in mammalian tissue.

### 2.4. Proofreading effort

Tolerant-edit distance (Funke et al., 2017) and estimates of focused proofreading correctness time (Plaza, 2016) provide another mechanism to measure segmentation quality. Good segmentation should require few proofreading corrections (shorter edit distance) than bad segmentation. A segmentation that splits a neuron in half would be better than one with several smaller splits, since the former would only require one merge and the later several mergers. Designing interpretable edit distance formulations are challenging because different proofreading workflows could lead to very different proofreading reconstruction times.

The usefulness of the above metrics often depend on the application. For practical reasons, mathematically well-formed metrics like VI and ERL that have few parameters are often favored. Metrics that better reflect connectivity are harder to define since they depend more on the target application or require the existence of synapse annotation which is currently predicted in a separate image processing step from segmentation.

Finally, there has been only limited exploration in using segmentation metrics as debugging tools. Presumably, this becomes a bigger concern when evaluating larger datasets. Notably, the authors in Reilly et al. (2017) recognized this challenge and describe a metric that allows intuitive insights at the neuron level. In Nunez-Iglesias et al. (2013), the authors decompose the VI calculation to provide scores per 3D segment. For instance, the over-segmentation VI score *H*(*S*|*G*) can be decomposed as a sum of oversegmentation per ground truth neuron *g*:

(2) $$\begin{array}{r} H\left(S|G\right)=-\underset{g}{\sum }P\left(g\right)H\left(S|G=g\right) \end{array}$$

Presumably, other metrics like ERL, can be used to provide neuron-level information for finding the worst segmentation outliers.

## 3. Metric evaluation ecosystem

We introduce a metric evaluation ecosystem that is designed to assess the quality of large, practical-sized datasets. To this end, we propose evaluation paradigms that emphasize interpreting and debugging segmentation errors that make comparisons between two different segmentations. While having ground truth is mostly necessary to quantify whether one segmentation is better than another, meaningful comparisons are possible without laboriously generated ground truth since the metrics highlight differences and these differences can be readily inspected. In the following few paragraphs, we will discuss the overall philosophy of our efforts. Then we will explore in more detail novel metrics and the software architecture.

In this work, we do not advocate a specific metric, but instead recommend an “all-of-the-above” framework where for each dataset multiple metrics are used to provide different subtle insights on segmentation quality. While not every popular metric is implemented, our framework is extensible and can support customized plugins.

We provide feedback on segmentation quality at different levels of granularity: summary, body, and subvolume.

### 3.1. Summary

Each segmentation sample is evaluated with several scores applied to the whole dataset. These scores do not provide insight to where errors occur but provide a simple mechanism to compare two segmentation algorithms succinctly. VI and Rand index are two such examples. Section 3.4 introduces several new connectivity-based metrics.

### 3.2. Body

We provide per segment (or body) statistics with respect to segments from both datasets *S* and *G* (*G* need not be ground truth). For example, this includes the per-body VI score defined in Equation 2, which provides insights on where over and under-segmentation occur in the volume. We highlight a couple new body metrics in section 3.4.

### 3.3. Subvolume

When appropriate, metrics that are computed for the whole dataset are also applied to a regular grid of subvolumes that partition it. In this manner, the quality of segmentation can be assessed as a function of its location in the volume. This is useful for potentially detecting regions in the dataset where a classifier fails to generalize. For example, the framework runs VI on each subvolume. To partially disambiguate errors that originate in one region but propagate to another, distant region, we apply a local connected component algorithm to treat each subvolume as an isolated test segmentation^1^.

**Figure 3:**
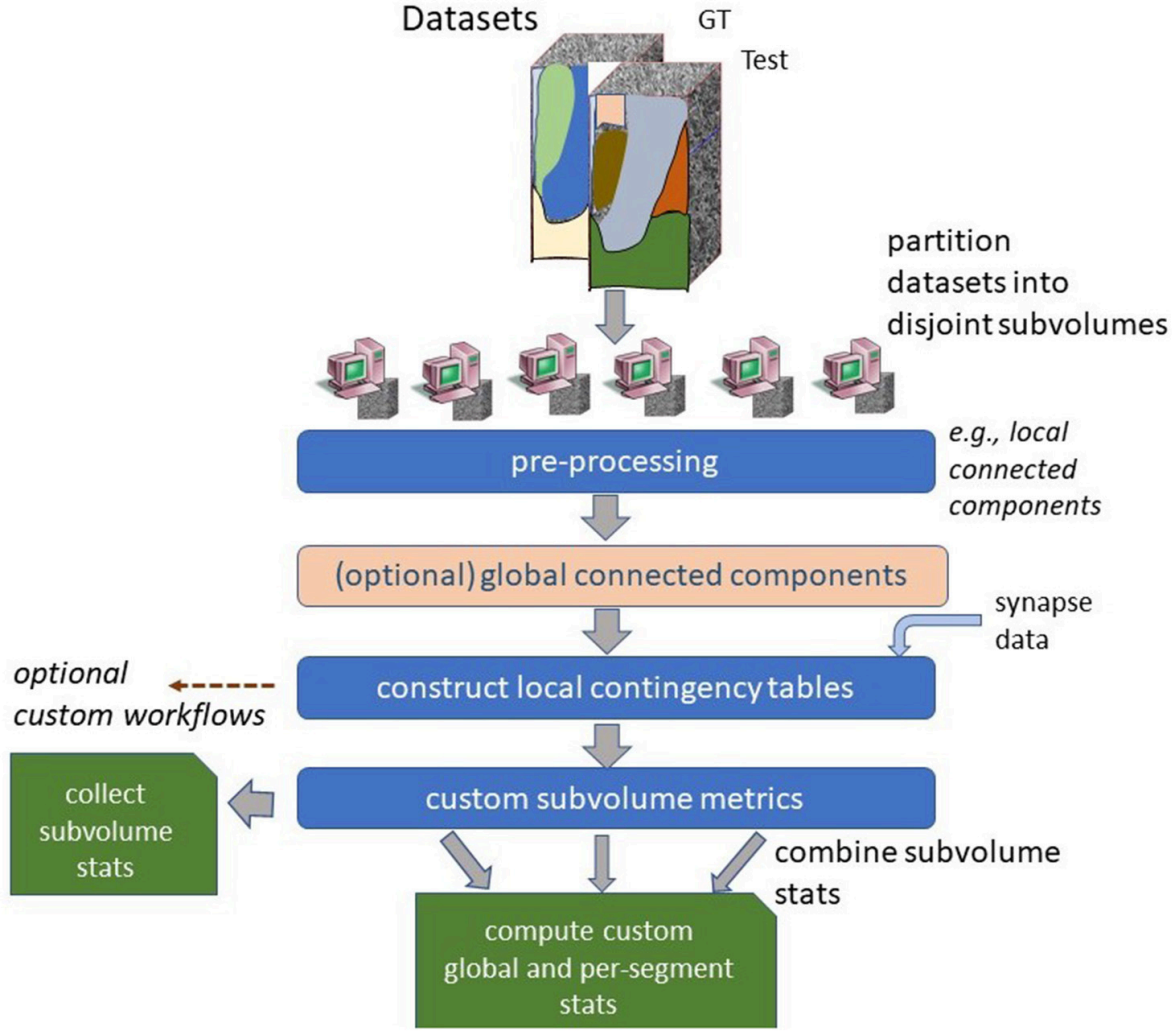
High-level parallel evaluation framework. Segmentation and ground truth data is partitioned into several small pieces. Most metrics are computed in parallel by combining local contingency tables.

The evaluation framework can run over several distinct sets of comparison points. By default, segmentations are compared at the voxel level, i.e., the comparison points are all segmented voxels. If other sets of *important* points (such as synapses) are provided, analysis is similarly applied over these sets. The evaluation provides a mechanism to compare against oneself (no ground truth or alternative segmentation). We discuss metrics that enable self-evaluation in Figure 3. Comparisons to ground truth can be restricted to sparsely reconstructed volumes or dense labeling.

### 3.4. Metrics

In the following, we highlight a few novel metrics for evaluating segmentation, which is a subset of all metrics implemented in the framework. These new metrics are divided into the categories of summary, per-segment, and self-comparison.

#### 3.4.1. Summary

We propose a metric to assess the connectivity correctness (CC) of the given segmentation *S* compared to ground truth *G*. At a high level, *CC*(*S*|*G*) defines the percentage of connections that match the ground truth connections. A connection is defined as an edge between two segments (neurons) that represents a synapse. There can be multiple connections between the same two segments. More formally:

(3) $$CC(S|G)=\frac{\sum_{(g_{i},g_{j})\in G}\left|x(A_{S}(g_{i}),A_{S}(g_{j}))\cap x(g_{i},g_{j})\right|}{\sum_{(g_{i},g_{j})\in G}\left|x(g_{i},g_{j})\right|}$$

where *x* returns the set of synapse connections between two segments. *A*_*S*_(*g*_*i*_) determines the optimal assignment of groundtruth segment *g*_*i*_ to a segment in *S* (e.g., using the Hungarian matching algorithm). The matching is one-to-one and if there is no match *x* will be an empty set. In practice, an algorithm that greedily finds a set of matches by using greatest segment overlap with ground truth is likely sufficient since one would not expect the set of intersecting segments in *S* to a given segment in *G* to greatly overlap with intersection sets to other segments in *G* in a manner that would require joint optimization. This is true by construction in the scenario where every segment in *S* is either a subset of a given segment *G* or equal to a set of *g*.

This metric is sensitive to both false merge and false split segmentation errors. If there is a false split, there will be fewer matching connections compared to ground truth. If there is a false merge between *g*_1_ and *g*_2_, the one-to-one assignment *A*_*S*_ ensures that *A*_*S*_(*g*_1_) ≠ *A*_*S*_(*g*_2_) meaning that there will be no matching connections involving either *g*_1_ or *g*_2_.

Additionally, we introduce a thresholded variant of the connectivity metric to emphasize the percentage of connection paths that are found with more than *k* connections. We modify Equation 3 to include this threshold and decompose into recall and precision components as defined below:

(4) $$recCC_{k}(S|G)=\frac{\sum_{(g_{i},g_{j})\in G}I(|x(A_{S}(g_{i}),A_{S}(g_{j}))\cap x(g_{i},g_{j})|>k)}{\sum_{(g_{i},g_{j})\in G}I(|x(g_{i},g_{j})|>k)}$$

(5) $$preCC_{k}(S|G)=\frac{\sum_{(g_{i},g_{j})\in G}I(|x(A_{S}(g_{i}),A_{S}(g_{j}))\cap x(g_{i},g_{j})|>k)}{\sum_{(s_{i},s_{j})\in S}I(|x(s_{i},s_{j})|>k)}$$

The above metrics to measure the similarity between two connectomes have advantages over using a more general graph matching algorithm. First, by requiring an initial assignment of each segment to a groundtruth neuron (if a distinct match exists), the CC metric aims to better constrain the problem of measuring the similarity between two connectivity graphs, thereby avoiding the need for the computational complexity typical in general graph matching algorithms. Second, the CC metric allows one to express the matching in terms of individual neurons and number of connections preserved, which is more biologically intuitive compared to a general edit distance score.

In addition, to *CC*_*k*_, we define a class of statistics that analyzes the fragmentation of *S* compared to *G* based on the simple formula:

(6) $$\begin{array}{r} Frag=|S|-|G| \end{array}$$

where a high score indicates that *S* consists of many more segments than *G*. While very simple, this provides a lower-bound on the number of edits (or segments to “fix”) to transform *S* into *G*. In practice, we find that *S* is typically an over-segmented subset of *G* and *Frag* provides a reasonable edit distance estimate. We can extend *Frag* by extracting a subset of *S* and *G*, S* and G*, that represent a less-than-100% correct segmentation. More specifically, we define a thresholded fragmentation score, where S* and G* are the smallest set of segments whose cumulative size reaches a specified size threshold, where size can be number of voxels or synapses. This trivially computed measure allows us to discern the number of segments required to produce a connectome that is *X%* complete.

#### 3.4.2. Body

As described in Equation 7, VI can be decomposed to provide insight about the fragmentation of a given segment. If this score is applied with respect to segment *g*, it provides an over-segmentation score of *g*. If this applied with respect to segment *s*, it provides an under-segmentation score of *s*. We can alternatively decompose the VI calculation to report the over and under-mergers that intersect a given segment. We define the under and over segmentation score for *g* as:

(7) $$\begin{array}{r} H\left(S|g\right)+H\left(g|S\right)=-P\left(g\right)H\left(S|G=g\right)-\underset{s}{\sum }P\left(s\right)H\left(G=g|S=s\right) \end{array}$$

where *P* is the the probability of *g* (or percentage of *g* in *G*). This metric is useful to provide a simple score for the neuron that has the worst segmentation. This metric works most naturally over a densely labeled *G* since the impact of the false merging can be more accurately assessed.

Additionally, we modified the metric in Equation 3 to provide a score for each *g* the percentage of connections that are covered. We further note which bodies are the most correct by simple overlap, which is conceptually similar to examining the largest error-free run lengths often used in skeleton-based reconstructions.

#### 3.4.3. Self-compare

As mentioned, the ability to decompose the metrics at segment level allows one to compare two different segmentations. However, it is often useful to have some information on segmentation reliability when no comparison volume is available. One simple statistic that can be extracted is the number of segments that are needed to reach a certain volume threshold (as defined previously), which provide insights in regions that are relatively over-segmented compared to others. However, this metric can be misleading since neuropil regions vary in neuron packing density.

We introduce two metrics to better assess segmentation in the absence of ground truth: orphan segments and segmentation loops. Biologically, one does not expect a neuron to be a small fragment below a certain size *K*. A count of the number of segments below this threshold, provides a crude error measure. This will not uncover potential under segmentation errors. To find potential under segmentation errors, we note that neurons should have few connections to itself (self-loops). By counting the number of autapses or finding the segments that have a lot of autapses, we can detect potential false mergers. As segmentation gets better the effectiveness of using autapses as a proxy for false-merge errors is limited since such connections due exist in practice, such as in the *Drosophila* medulla connectome in Takemura et al. (2015). Therefore, the loop detector should be viewed as a mechanism to detect outliers due to either segmentation error or biological design and serve as a good entry point for analyzing a segmentation. Depending on the organism and the extent of the region being evaluated, additional metrics could be considered, such as ensuring that each segment has both inputs and outputs. We only formally consider orphans and self-loops in this work.

### 3.5. Architecture

We introduce an Apache Spark-based system for comparing two, large segmentations at scale. The implementation is built over the framework described in Plaza and Berg (2016) and is available at https://github.com/janelia-flyem/DVIDSparkServices as the EvaluateSeg workflow . The segmentation and synapse data is stored using DVID (Katz and Plaza, 2018). In general, segmentation compresses to a small fraction of the original EM data size and we do not observe fetching segmentation to be a bottleneck in the analysis workflow. However, evaluating on datasets that are significantly larger than the 1 gigavoxel datasets common in SNEMI and CREMI necessitates a framework that can compute metrics on a large-memory, multi-core, cluster environment.

An overview of the software workflow is shown in Figure 3. We partition the dataset into disjoint, equal-size subvolume for a region of interest (ROI). A local connected component algorithm is computed for each subvolume and other filters are applied, such as (1) dilating groundtruth segment boundaries to reduce the impact of small variations in the exact boundary between segmentation and (2) filtering out neurons that are not groundtruthed for sparse evaluation. If the ROI being analyzed is part of a larger segmentation, one can run a global connected component algorithm which ensures that segments that merge outside of the ROI are treated as separate objects within the ROI. The global connected component algorithm is computed by examining the boundaries between all subvolumes in parallel and determining which components have a connecting pathway through the ROI.

For each subvolume, we compute a contingency table between segments in *S* (when not doing a self-comparison) and *G* (where *G* is treated like ground truth unless otherwise specified). The overlaps computed between *S* and *G* allow many of the metrics to be computed per subvolume and then combined into global summary and body stats. This is done over the set of voxels and optionally any available synapse (or other point) data. In the current workflow, one of the largest, non-parallelized compute components is this final grouping of results. Future work to further reduce these non-parallel points is possible but not currently necessary for the experimented data sizes.

The framework allows additional plugins that conform to the API to be added without changing the surrounding framework. In circumstances where this partitioning and combination strategy will not solve a given metric algorithm, it is possible to define a completely custom workflow based on the input segmentation. The current framework does not implement ERL or other skeleton-based metrics, but our ecosystem should admit for its straightforward inclusion.

**Figure 4:**
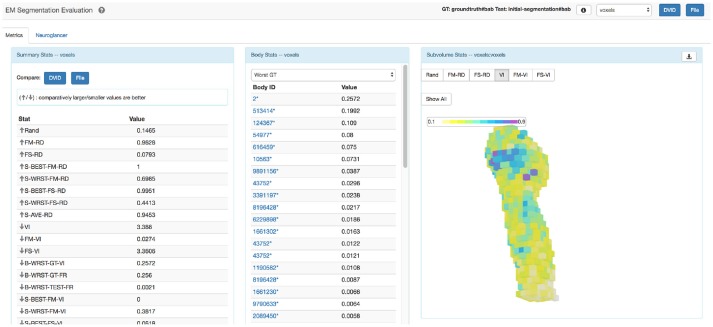
Evaluation web application. Web application that displays results and tools to visualize segmentation errors.

The statistics from this computation are collected into a file that can be easily parsed. However, the myriad of metrics can make interpreting results overwhelming, so we designed a single web page application in Javascript as shown in Figure 4 to improve accessibility. The web application groups similar stat types together displaying the list of summary stats and per-body breakdowns for provided metrics. A visualization tool shows a heat-map highlighting subvolume to subvolume variation in segmentation quality. The application also allows one to compare the summary results of two different segmentation evaluations. The web page application is available at https://github.com/janelia-flyem/SegmentationEvaluationConsole.

## 4. Experiments

We demonstrate our evaluation framework on two large, public datasets: a portion of the *Drosophila* medulla (Takemura et al., 2015) and mushroom body (Takemura et al., 2017). The medulla dataset segmentation and grayscale can be accessed at http://emdata.janelia.org/medulla7column, and the mushroom body dataset can be accessed at http://emdata.janelia.org/mushroombody. Both datasets are around 20 Gigavoxels in size and contain over 100,000 synaptic connections. Since neither dataset is 100 percent accurate, we filter small orphan segments in the ground truth using options in the metric tool and we dilate ground truth neuron boundaries with a radius of two pixels. We compare these ground truths to initial segmentation generated using a variant of the algorithm developed in Parag et al. (2015).^2^ A smaller portion of the optic lobe segmentation is also compared against a more recent segmentation algorithm (Funke et al., 2018). The purpose of the following experiments is to demonstrate the breadth of provided metrics, as well as, some insights that might impact how one analyzes segmentation results.

### 4.1. Summary results

**Figure 5:**
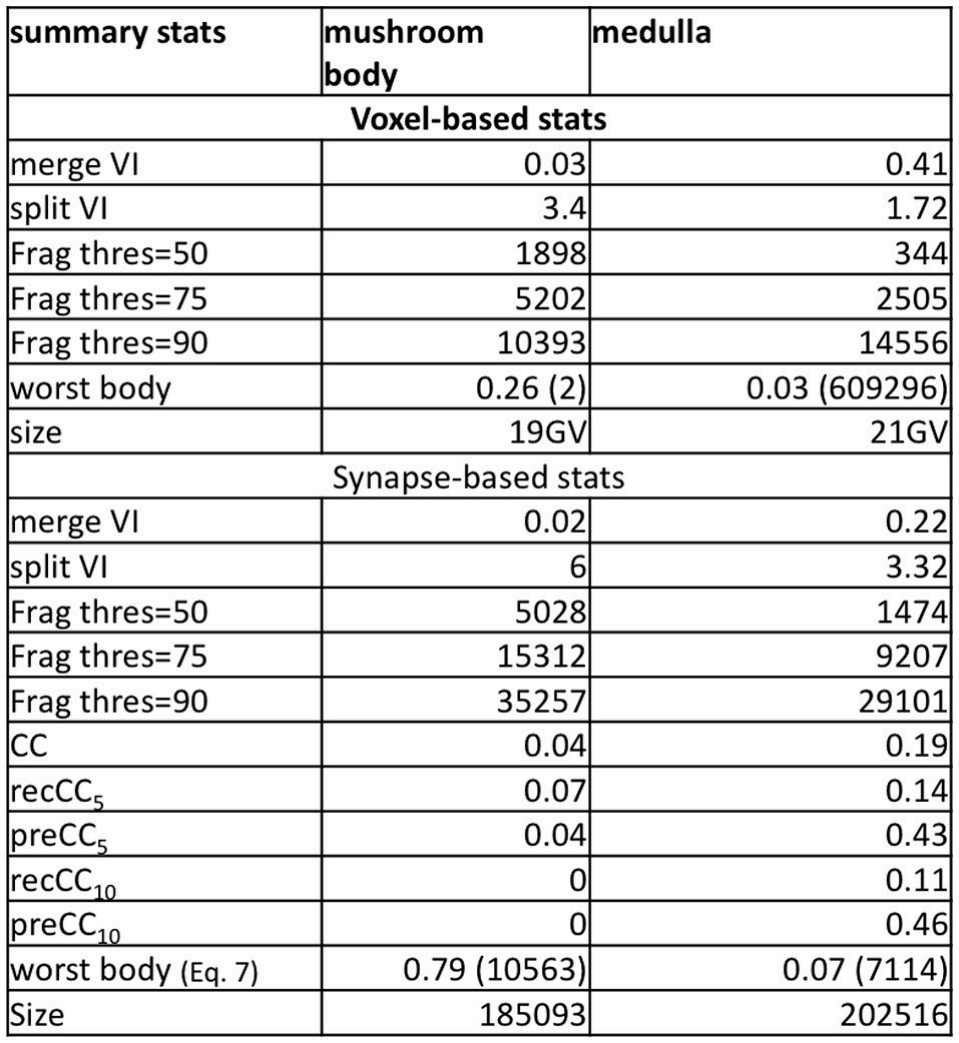
Select metrics for the medulla and mushroom body dataset. The data shows voxel-based metrics like VI and less-common, but more useful synapse-based metrics. The histogram metric shows the many more segments are required to reach X percent of the total volume.

The evaluation service produced a series of summary stats. A subset of these are depicted in Figure 5. The stats are split into two broad categories: voxel-based and synapse-based. The voxel-based stats provide volume-relevant information. The synapse-based stats emphasize only the exemplar points that define each input and output for a synapse.^3^

In both the mushroom body and medulla, we notice that there are very few false merge mistakes indicated by merge VI. Notably, the split VI is much higher when focusing near synaptic regions. The comparably higher values in the mushroom body highlight both the conservative segmentation used and the presence of very small, hard-to-segment processes. The thresholded segment count shows that to examine 50 percent of the synaptic points, a relatively small number of segments need to be examined compared to achieving 90 percent coverage. For both datasets, the connectivity correctness defined by Equation 3 is very low, in particular in the mushroom body where the neurites are very small. This indicates that the automatic segmentation is far from being useful for biological analysis without proofreading.

**Figure 6:**
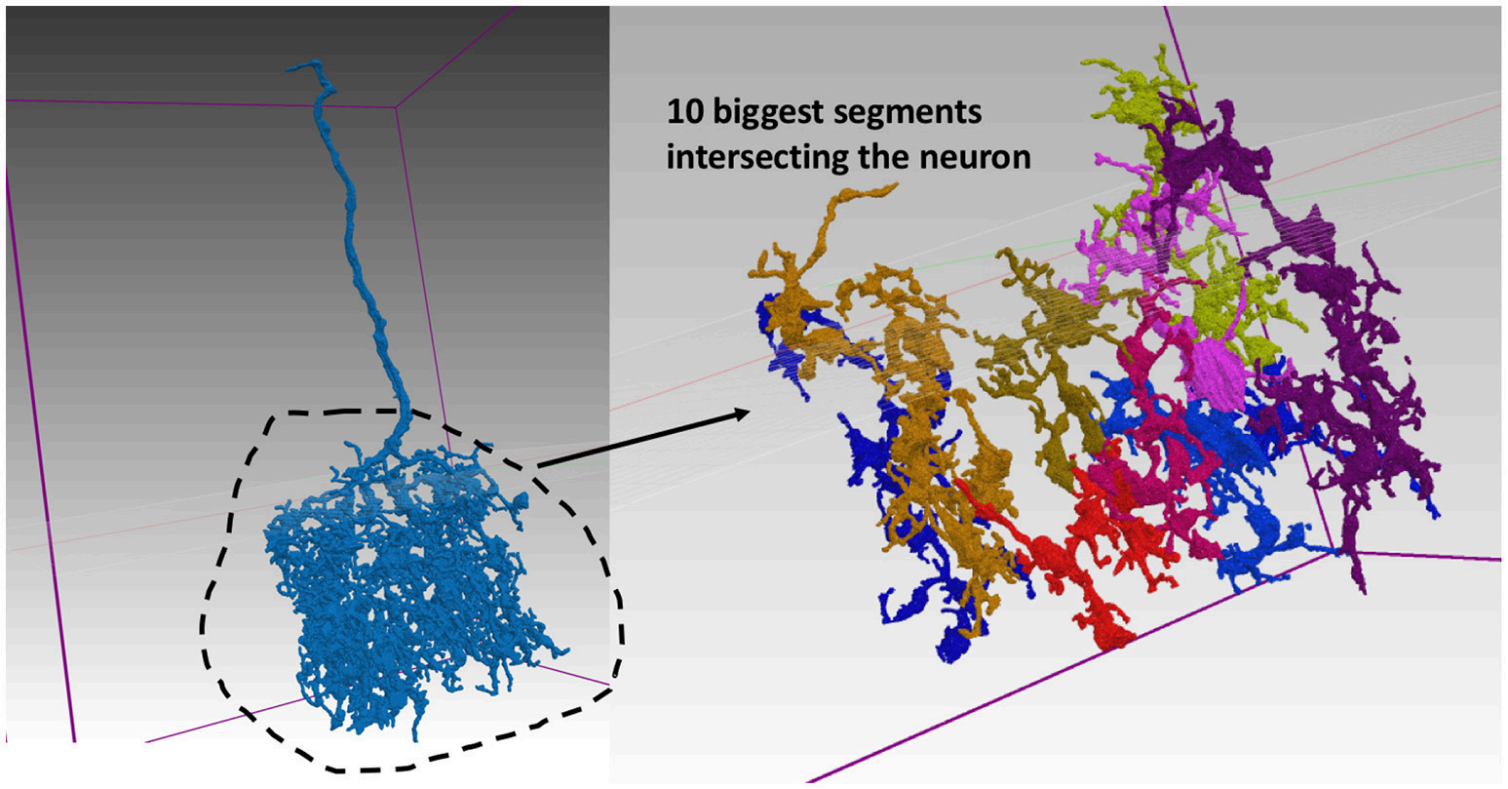
Segmentation for worst neuron in medulla. The neuron on the left segmented poorly according to the body-based synaptic VI metric. The top 10 largest pieces from the example segmentation are shown on the right and make up only a small portion of the neuron's complex arbor.

The summary results also report the worst body VI score and the segment ID number corresponding to this body. We show one example from the medulla in Figure 6. The evaluation service reports the biggest overlapping segments. Notice that the top 10 biggest fragments only cover a small portion of the complex neuron arbor.

**Figure 7:**
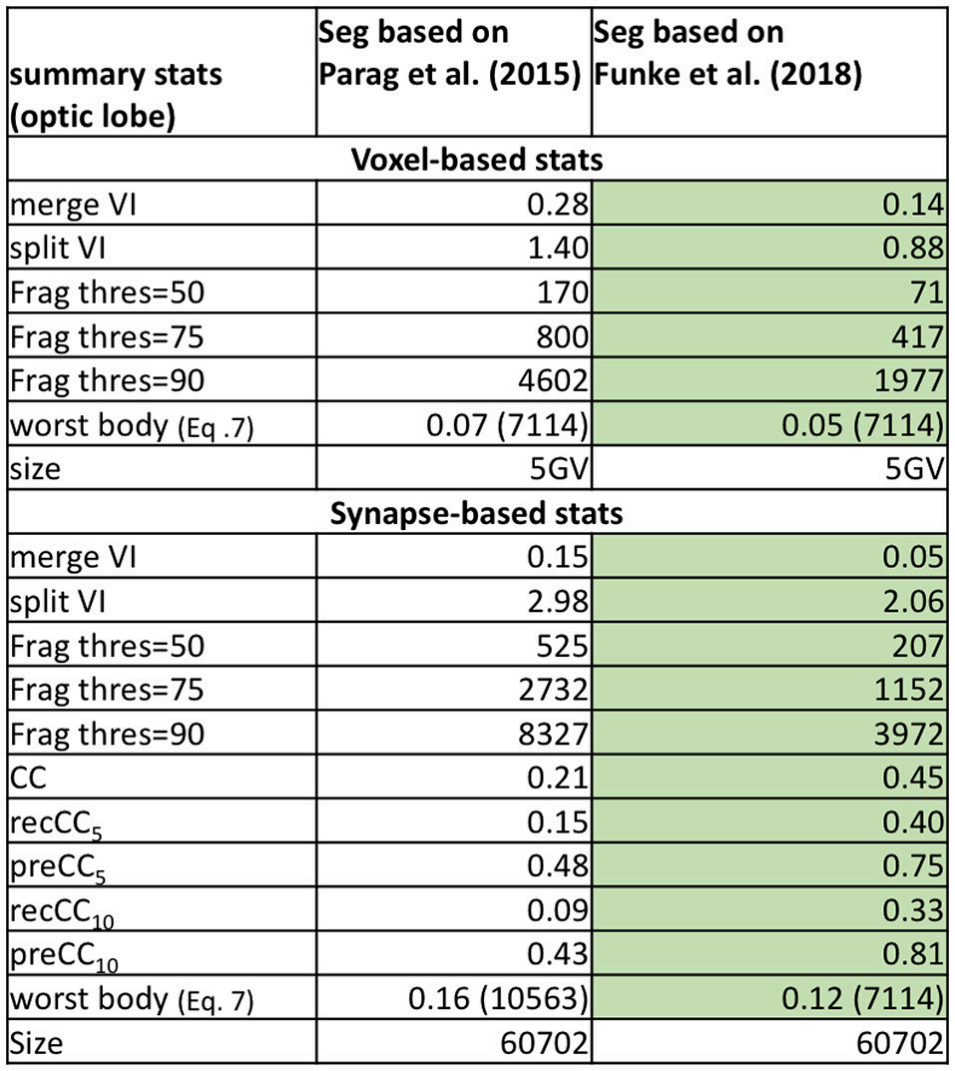
Comparing two segmentations from a subset of the medulla dataset. Unsurprisingly, the more recent segmentation from Funke et al. (2018) performs better on all metrics (indicated by the highlighted boxes). In particular, Funke et al. (2018) achieves much higher CC scores finding 33 percent of all neuron connections with weight greater than or equal to ten synapses, compared to only 9 percent for the baseline.

We compare the baseline segmentation with a newer segmentation approach in Funke et al. (2018) for a subset of the medulla dataset in Figure 7. As expected, Funke et al. (2018) achieves a better score across all reported metrics. While the VI scores indicate significant improvement, the fragmentation thresholds and synapse connectivity clearly show the advantages for the newer segmentation. There are far fewer segments to consider to reach different levels of completeness as seen in Frag thres. Perhaps more significant is the much greater percentage of neuron connections found with the new segmentation. The CC metrics are sensitive to large neurons being correct in addition to the small synapse processes being correctly segmented. Metrics less sensitive to this level of correctness, like the VI numbers reported, might, in effect, over-rate the quality of inferior segmentation.

### 4.2. Unsupervised evaluation

**Figure 8:**
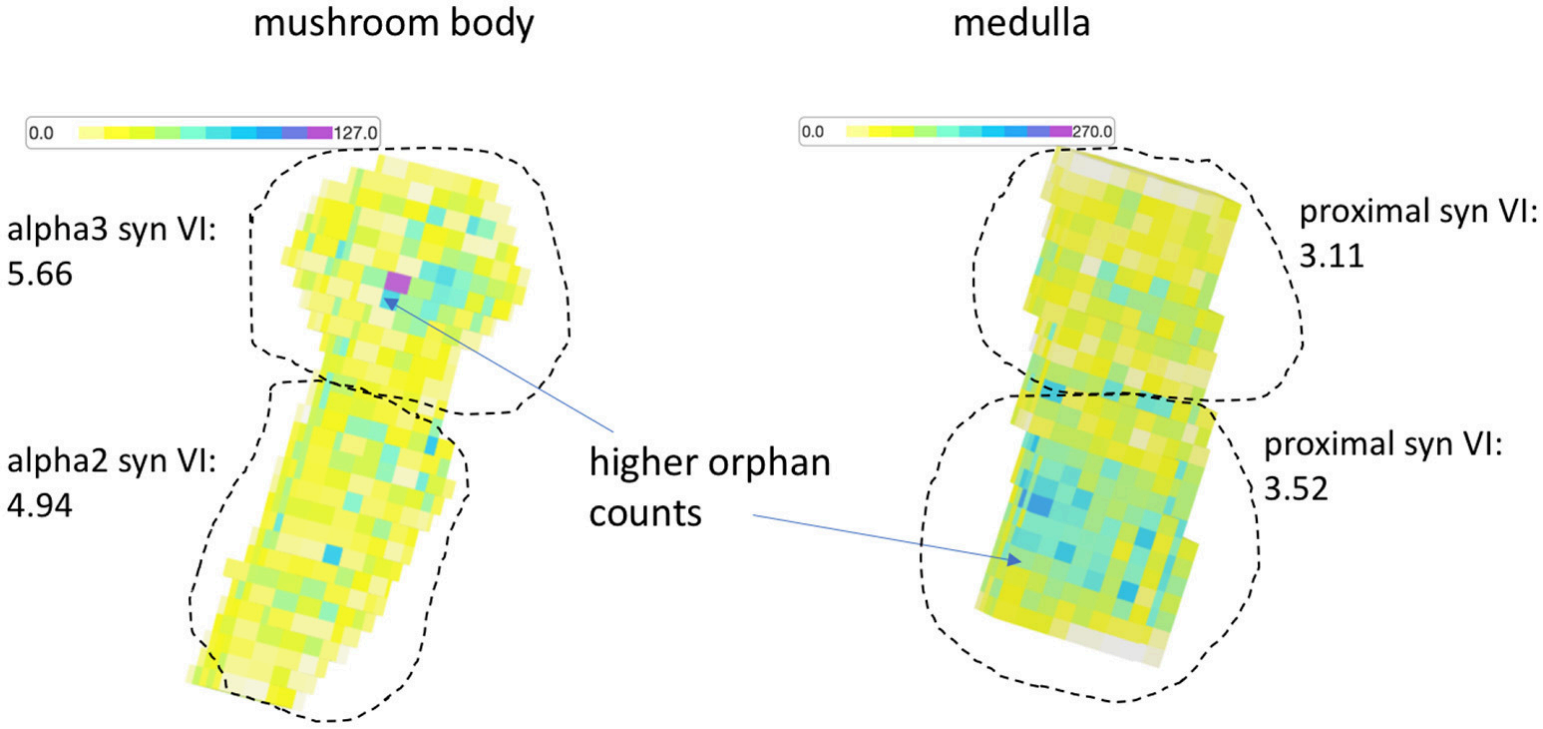
Orphan density map. For both the medulla and mushroom body sample, the orphan count density (an unsupervised statistic) appears greater (darker) in regions with worse synaptic VI compared to the other regions.

The previous results show comparisons between test segmentations and ground truth. As previously explained, the metric service is useful for comparing two segmentations directly even if one is not ground truth since there are many stats that highlight differences useful for debugging. For instance, while the VI between two test segmentations fails to suggest which one is better, it does indicate the magnitude of the differences, can indicate whether one segmentation is over-segmented compared to the other, and gives a list of bodies that differ the most, which can then be manually inspected to determine segmentation errors. But we also introduced stats that do not require a comparison volume. We evaluated both medulla and mushroom body in this way. In Figure 8, we see a heatmap highlighting the small orphan segmentation density over the subvolumes that partition both datasets. We define orphan as any segment with fewer than 10 synaptic endpoints. Visually, the diagram shows more errors in the alpha 3 lobe and proximal region of the mushroom body and medulla respectively. If we evaluate these regions separately against the ground truth, we observe that the supervised VI scores are consistent with the unsupervised visualization.

We were also able to find one neuron in the medulla dataset that had many autapses, which suggests a potential false merge. This worst neuron in the un-supervised analysis corresponds to the fourth worst body in the supervised analysis. This suggests that the autapse count can reveal false merge errors.

### 4.3. Performance and scaling

These datasets are much larger than previous challenge datasets but are still much smaller than the tera to peta-scale datasets that are being produced. One obvious solution to handling larger datasets is to run the framework on a larger compute cluster.

**Figure 9:**
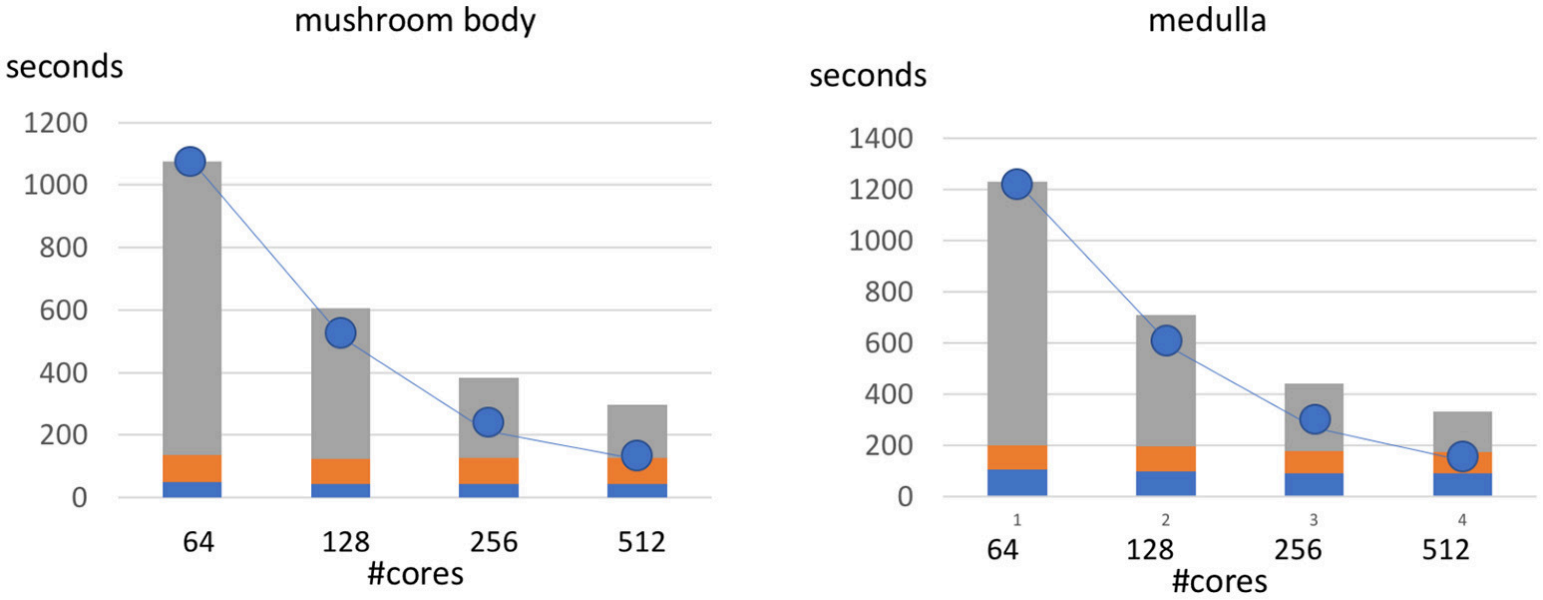
Runtime of metric computation at different levels of parallelization. The line represents the optimal speedup for increasing the number of cores from the baseline 64 core implementation. The non-parallelized part of the framework represents the computation performed solely on the driver node and is indicated by the bottom two sections of each bar. This non-parallel time is around 129 and 187 s from the mushroom body and medulla respectively.

We show the scalability of our framework by evaluating our two sample datasets with varying numbers of cores. The charts in Figure 9, shows a breakdown of runtime between the top parallelizable portion of the code and the bottom, sequential small overhead. As the number of cores increase we observe a speedup that is slightly less than linear to the number of added cores (indicated by the trendline). We observe that the sequential overhead indicated by the lowest two section of each bar is roughly constant and a small portion of this time (the lowest section) could potentially be partially parallelized with future optimizations.

The results in the table suggest that 512 cores can roughly process around 20 gigavoxels in around 5 min, or over 60 megavoxels of data per second, or 1 TB in a little over 4.5 h. Note that the comparison framework requires two datasets to be processed and this analysis includes the global connected components analysis, which is not necessary if segmentation is completely contained within the defined region. Also, note that medulla and mushroom body ROIs do not perfectly intersect the subvolumes, so more data is actually fetched to retrieve the entire 20 gigavoxel ROI.

In practice, we expect additional bottlenecks if there are a lot of small segment fragments which could lead to more computation in the sequential parts of the code and in shuffling data around on the network. Future work should aim to improve the performance when dealing with a large number of small fragments since its relevance to analysis is mostly in the aggregate and not at the individual fragment level. We do not observe slowness fetching the segmentation data, but the data could always be partitioned between multiple servers to allow for higher cumulative read bandwidth.

**Figure 10:**
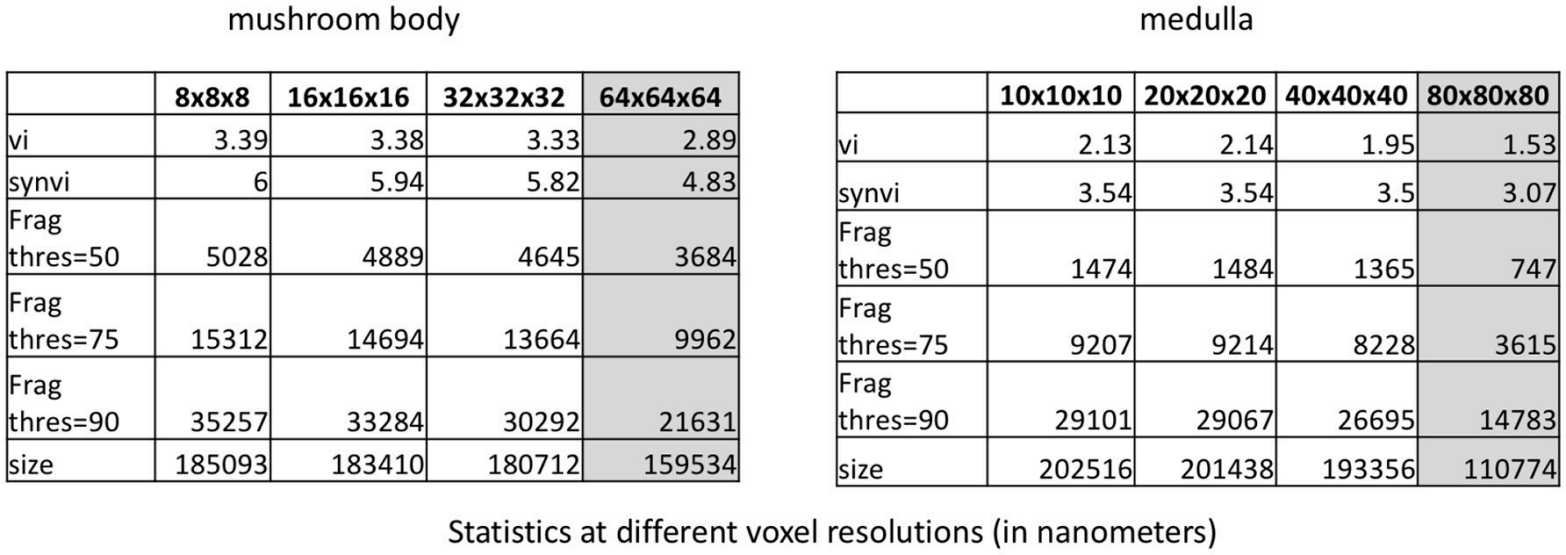
Stability of various metrics when downsampling the dataset. When the voxel resolution is higher than 40 × 40 × 40 nm, the results are fairly consistent. When the voxel resolution is too low, several synapses on smaller neurites are missed. The 50, 75, and 90% connections number refers to the number of segments required to cover the specified percentage of connection endpoints.

To further improve performance, we consider downsampling the segmentation. (A multi-resolution segmentation representation is available in DVID and does not need to be computed.) Figure 10 shows both datasets at original resolution and downsampled by a factor of 2, 4, and 8 along each axis. One might expect that downsampling the dataset considerably would greatly change the statistics particularly related to fragmentation due to presumably small synaptic processes. Perhaps surprisingly, a few key metrics have a consistent value when downsampling by 4x suggesting that significant computation reduction is possible since full resolution is unnecessary. For example, the fragmentation scores in these datasets, which provide a rough estimate of the number of merge edits required, is similar (within 20 percent) to full resolution. Once the resolution starts getting worse than 40x40x40nm, there is considerable impact on the synaptic VI and the number of thresholded segments. However, the significant differences reported between the two segmentations in Figure 7 are preserved even at the lowest resolution tested.

## 5. Conclusions

In this work, we demonstrate a metric evaluation framework that allows one to analyze segmentation quality on large datasets. This work necessitated diverse contributions: new metrics that provide novel insights in large connectomes, a software framework to process large datasets, and visualization software to enable intuitive consumption of the results. All of these contributions, in synergy, were critical to enable segmentation evaluation in practical settings.

We implemented multiple metrics to provide different insights on segmentation. In particular, we introduced new connectivity-based metrics that clearly show that significant improvements are still needed to produce fully-automatic reconstructions, which seem to correctly reflect our observations in practice. Furthermore, we note that for purposes of comparison, it is possible to downsample the data significantly without significant impact on important metrics. Finally, we introduced the possibility of comparing two segmentations without ground truth, where evaluation can be done by manually inspecting the largest segmentation differences revealed by decomposing the metrics in different ways and providing useful visualizations, such as showing segmentation quality variation as a function of region location. We believe that this work should help accelerate advances in image segmentation algorithm development and therefore reduce bottlenecks in large connectomic reconstructions.

The diverse set of statistics produced by our workflow could make the task of comparing segmentations overwhelming, as one desires to know which is the best metric. This paper has taken an agnostic position to the best metric largely because it depends on the application. If one is concerned about optimizing proofreading performance, edit distance measures make the most sense. However, this is complicated because edit distance costs depend on the proofreading methodology. The fragmentation scores provide a very intuitive, parameter-free measure of segmentation quality if one has mostly tuned the algorithms to over segment, since the number of segments is a guide for the number of mergers required. To assess whether the segmentation can be used in a biologically meaningful way, our new connectivity metric will provide the best insight on the quality of the resulting connectome. For assessing general neuron shape correctness, ERL (which we do not currently implement) or VI can be used.

We expect additional improvement is needed to further parallelize sequential portions of the framework. Also, we believe that additional metrics should be invented that provide interesting insights for evaluating the connectivity produced from the segmentation. We have introduced a few metrics to this end in this paper. We advocate the inclusion of more metrics in evaluation to better understand the failure modes of segmentation, which will hopefully lead to the implementation of better algorithms.

## Author contributions

SP devised and implemented the core methodology, JF contributed some image segmentation and metric discussions.

### Conflict of interest statement

The authors declare that the research was conducted in the absence of any commercial or financial relationships that could be construed as a potential conflict of interest.

## Footnotes

^1^In cases of serious false merging that results in incidental contact between segments far away from the error site, connected components within a subvolume containing this site will be ineffective.

^2^We only have an archival version of mushroom body segmentation available where a few catastrophic false mergers were already eliminated.

^3^In *Drosophila* tissue, synaptic connections are polyadic meaning multiple post-synaptic targets for one pre-synaptic region. For this analysis, each pre and post-synaptic site represented by a single exemplar point is considered as one connection endpoint.
